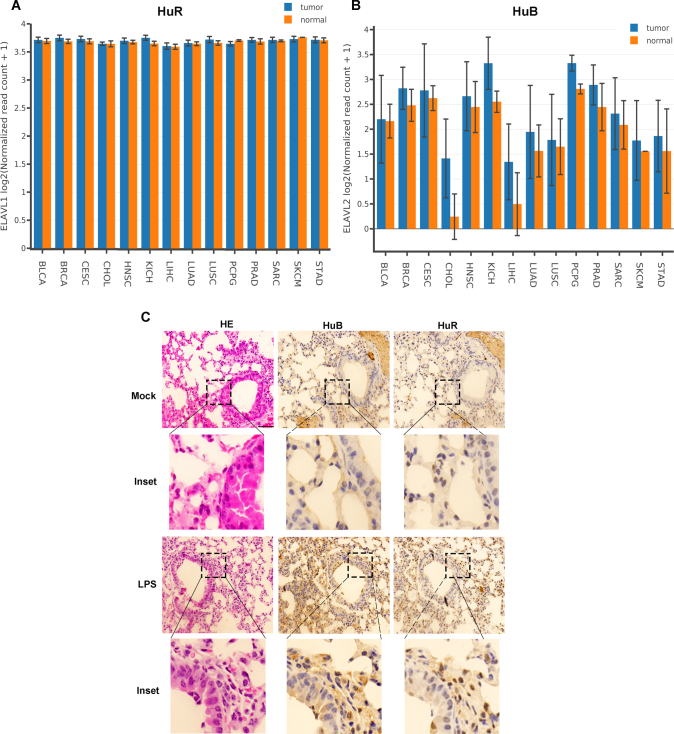# HuR-HuB autoregulatory network governs inflammatory factors expression

**DOI:** 10.1016/j.jbc.2026.113175

**Published:** 2026-05-20

**Authors:** Xingyue Fu, Yuhan Lu, Leying Kong, Yijun Qi, Xiyue Zhong, Xueqing Ba, Yueshuang Ke

**Affiliations:** Key Laboratory of Molecular Epigenetics of the Ministry of Education, School of Life Science, Northeast Normal University, Changchun, Jilin, China

**Keywords:** HuB, HuR, mRNA stability, inflammatory factors, post-transcriptional regulation

## Abstract

Short-lived mRNAs harbor AU-rich elements (AREs) in their 3′ untranslated regions (3′UTRs) that are tightly regulated by ARE-binding proteins. While the Hu family stabilizes target mRNAs, our understanding is largely confined to HuR due to its ubiquitous expression. Other Hu members, long considered neuron-specific, remain incompletely characterized in non-neuronal cells. Notably, HuB is markedly upregulated in various tumors and under cellular stress, but the molecular mechanisms and functional significance underlying its upregulation remain elusive. Here, we show that inflammatory stimulation induces cytoplasmic translocation of HuR. HuR binds directly to the 3′UTR of HuB mRNA, thereby enhancing HuB mRNA stability. Upregulated HuB binds to the HuR nucleocytoplasmic shuttling sequence (HNS) through its RNA recognition motif 3 (RRM3) domain, thereby retaining HuR in the cytoplasm. Collectively, HuB and HuR form a heteromeric complex that coordinately regulates the stability of inflammatory factor mRNAs. Our study identifies HuB as a key post-transcriptional regulator of inflammatory genes and highlights the critical role of the HuB-HuR regulatory network in modulating the stability of inflammation-related mRNAs.

Messenger RNAs (mRNAs) that encode central regulators of signal transduction, inflammation, anti-apoptosis, and oncogenesis are intrinsically unstable (1, 2, 3). To maintain low basal abundance, these transcripts are governed by post-transcriptional circuits that converge on AU-rich elements (AREs) located within their 3′ untranslated regions (3′UTRs) (4, 5). AREs act as binary switches and serve as high-affinity binding platforms for a cohort of ARE-binding proteins (ARBPs). Most ARBPs, including AUF1 and TTP, trigger the rapid deadenylation of mRNAs (6, 7, 8). In contrast, embryonic lethal abnormal vision-like (ELAVL/Hu) proteins stabilize ARE-containing mRNAs (8, 9, 10). In response to diverse external stimuli, Hu family members enhance their ability to compete with other ARBPs for binding to ARE-containing mRNAs (11, 12). They achieve this either by altering their subcellular localization or by adjusting their protein expression levels (9, 13). This competitive binding stabilizes target mRNAs and promotes their translation. Ultimately, this process exerts critical regulatory effects on target gene expressions in pathological contexts, including inflammation, autoimmune diseases, and tumors (13, 14).

The Hu family comprises four members: HuR, HuB, HuC, and HuD. HuR is the most intensively studied owing to its ubiquitous expression. Diverse stress stimuli promote its nuclear-to-cytoplasmic translocation, where it stabilizes target mRNAs (9, 10, 11). In contrast, although HuB was the first cloned member of the Hu family, it has long been considered neuron-specific, a view that has constrained functional analyses of HuB in other cells and tissues. Recent transcriptomic studies, however, have expanded our understanding of HuB’s functions by detecting its expression in various tumor types (15, 16, 17). Using UV cross-linking and PAR-CLIP, Castello *et al.* mapped extensive HuB-RNA interactomes in HeLa cells, implicating HuB in the regulation of mRNA stability in cancer (15, 17). We further showed that inflammatory stimuli upregulated HuB, which recruits the helicase DHX9 to accelerate the translation initiation of cytokine mRNAs (18). However, how HuB maintains high expression under inflammatory stimulation and regulates downstream inflammatory responses remains to be elucidated.

In the present study, we reveal the molecular mechanism underlying HuB upregulation under inflammatory stimulation and explore the critical role of the HuB-HuR complex in the regulation of inflammatory gene expression. Mechanistically, under stress stimulation, HuR shuttles to the cytoplasm, binds to and stabilizes HuB mRNA, thereby driving the overexpression of HuB. HuB not only facilitates the cytoplasmic accumulation of HuR through direct interaction but also forms heterodimers with HuR to enhance the regulation of target mRNA stability. Together, these findings reveal that HuB is not only a regulated molecule but also an active regulator that interacts with HuR and ultimately works in synergy to stabilize the mRNAs of inflammatory factors. These findings provide a theoretical basis for the subsequent development of anti-inflammatory or anti-cancer therapies.

## Results

### HuR upregulates HuB expression at the post-transcriptional level under inflammatory stimulation

**Figure 1:**
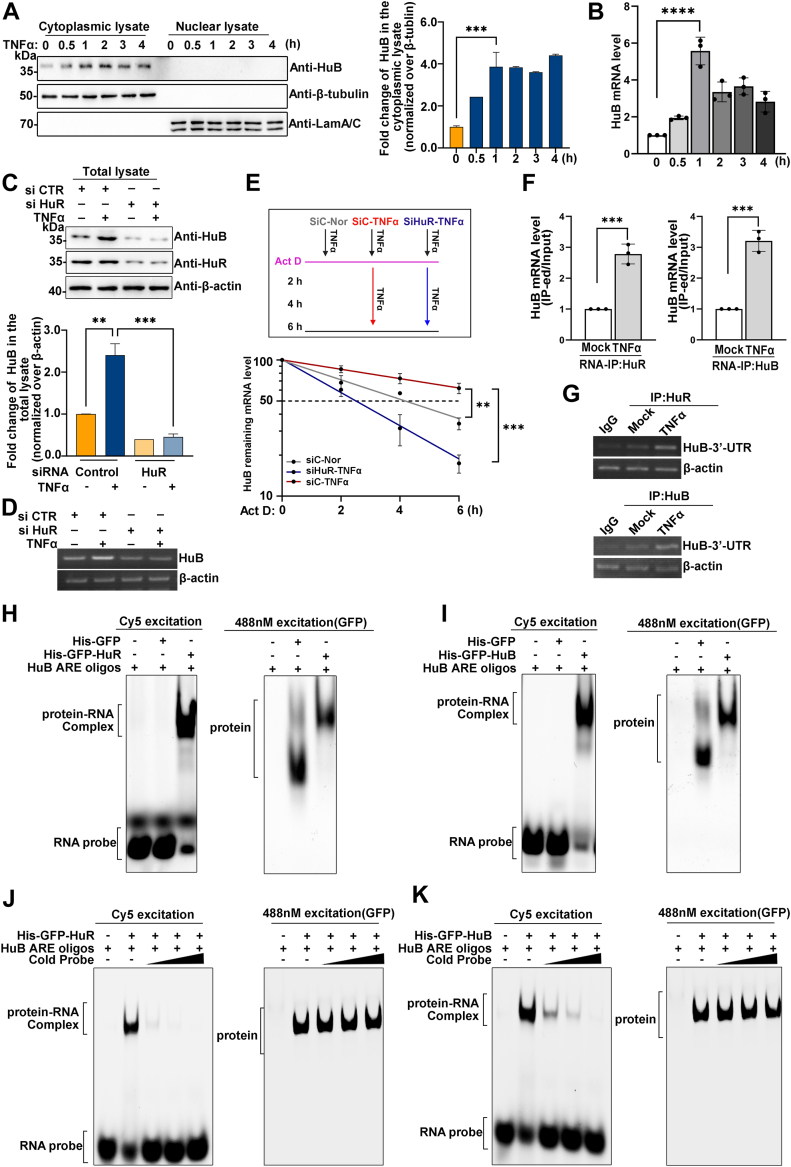
**HuR binds to the 3′UTR of HuB mRNA and upregulates HuB expression under inflammatory stimulation.***A* and *B*, inflammatory stimuli upregulate HuB expression. HEK293 cells were treated with TNFα for 0, 0.5, 1, 2, 3 and 4 h. Cytoplasmic and nuclear lysates were extracted, and immunoblotting was performed to detect the expression and subcellular localization of HuB (*left panel of A*). Quantitative analysis of the immunoblot results is shown in the *right panel of A*. Meanwhile, total RNA was isolated from HEK293 cells at the above time points after TNFα stimulation. Following reverse transcription, real-time PCR was conducted to measure the mRNA level of HuB (*B*). *∗∗∗p* < *0.001*, *∗∗∗∗p* < *0.0001*. *C* and *D*, HuR knockdown down-regulates HuB protein and mRNA levels. HEK293 cells were transfected with siRNA targeting endogenous HuR or si-control (si-CTR). After 48 h of transfection, the cells were treated with TNFα for 1 h. Total cell lysates were extracted, and immunoblotting was performed to detect the expression levels of HuR and HuB (*upper panel of C*), with quantitative analysis of the immunoblot results shown in the lower panel of *C*. Meanwhile, total RNA was isolated from the cells, and following reverse transcription, PCR was conducted to detect the mRNA level of HuB (*D*). *∗∗p* < *0.01*, *∗∗∗p* < *0.001*. *E*, Knockdown of HuR markedly reduces the half-life of HuB mRNA. HEK293 cells were transfected with siRNA targeting endogenous HuR or si-CTR. After 48 h of transfection, cells were pretreated with TNFα for 20 min, followed by treatment with actinomycin D (Act D, 10 μg/ml). HuB mRNA levels were quantified by real-time PCR at the indicated time points in groups with or without continuous TNFα stimulation (*upper panel*), and mRNA half-lives were calculated (*lower panel*). siC, siControl. *∗∗p* < *0.01*, *∗∗∗p* < *0.001*. *F*, endogenous HuR and HuB bind to HuB mRNA. Native RNA-IP was performed using anti-HuR, anti-HuB or control IgG antibodies in HEK293 cell lysates. The precipitated HuB mRNA was then quantified by real-time PCR. *∗∗∗p* < *0.001*. *G*, HuR and HuB binding to the 3′UTR of HuB mRNA. HEK293 cells were exposed to TNFα, then cross-linked RIP was conducted using anti-HuR, anti-HuB or control IgG antibodies. The bead-antibody-protein/mRNA complexes were subjected to PCR to detect the specific binding sites of HuB. *H* and *I*, HuR and HuB bind to the ARE region within the HuB 3′UTR. Purified His-GFP, His-GFP-HuR (*H*), or His-GFP-HuB (*I*) was incubated with the Cy5-labeled ARE sequence of HuB 3′UTR, and the protein-probe interaction was determined by RNA-EMSA. Specifically, the Cy5-labeled probe was used to detect the protein-probe complexes and the free probe, while GFP signals were used to verify the presence of the target proteins. *J* and *K*, HuR and HuB specifically bind to the ARE sequence of HuB. Purified His-GFP-HuR (*J*) and His-GFP-HuB (*K*) were incubated with the Cy5-labeled ARE sequence of the HuB 3′UTR. Subsequently, 10-fold, 20-fold, and 50-fold excess unlabeled HuB-ARE probes were added to perform cold probe competition assays. Specifically, Cy5-labeled probes were used to detect protein-probe complexes and free probes, while GFP signals were used to verify the presence of the target proteins.

**Fig. S1:**
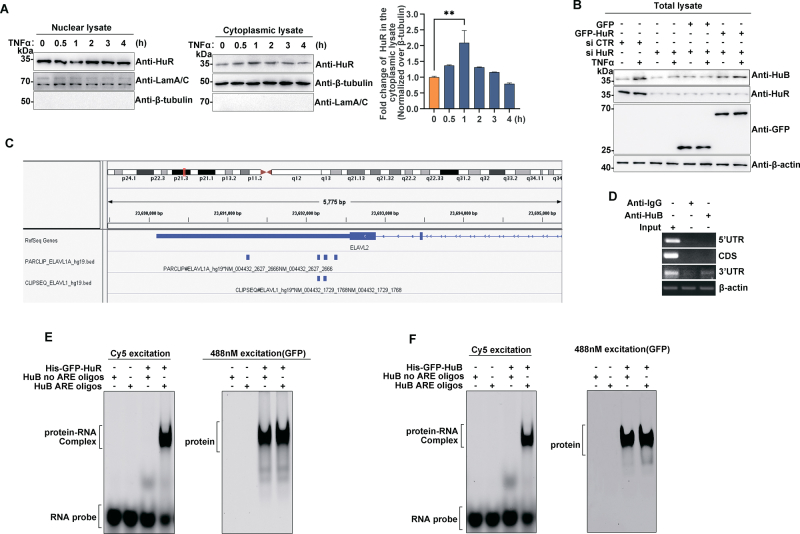


TNFα stimulation significantly upregulated HuB expression at both the mRNA and protein levels in HEK293 cells (Fig. 1, *A* and *B*). We also observed that the time window of HuR cytoplasmic translocation under inflammatory stimulation was highly consistent with the upregulation of HuB mRNA (Fig. S1*A*). Previous studies demonstrated that a core characteristic of the Hu protein family is the ability to autoregulate its own expression at the post-transcriptional level in response to extracellular stimuli (19), suggesting that post-transcriptional regulation may play a critical role in controlling HuB expression. Knockdown of HuR in TNFα-stimulated cells significantly decreased HuB protein levels (Fig. 1*C*). To exclude the possibility that the HuR siRNA sequence non-specifically interfered with HuB expression, we overexpressed GFP-HuR in cells with efficient HuR knockdown. The results showed that ectopic expression of GFP-HuR rescued the reduction in HuB expression caused by endogenous HuR depletion, confirming the target specificity of the si-HuR sequence and further demonstrating that HuR regulates HuB expression (Fig. S1*B*). Meanwhile, we observed that HuB mRNA levels were also markedly downregulated following efficient HuR knockdown (Fig. 1*D*), further suggesting that HuR may participate in the post-transcriptional regulation of HuB expression by modulating HuB mRNA stability. To directly verify this hypothesis, we examined the decay kinetics of HuB mRNA. Cells were pretreated with TNFα for 20 min, followed by the addition of actinomycin D (Act D) to block *de novo* transcription, and residual HuB mRNA was measured at successive time points (Fig. 1*E*). Compared with that in the control group, the degradation rate of HuB mRNA was significantly slower in the continuous TNFα treatment group (Fig. 1*E*). Moreover, HuR interference further shortened the half-life of HuB mRNA, directly confirming that HuR stabilizes HuB mRNA (Fig. 1*E*). Based on these findings, we conclude that the elevation of HuB mRNA under inflammatory stimulation relies largely on post-transcriptional regulation, even though transcriptional regulation cannot be completely excluded, and that HuR-mediated stabilization of HuB mRNA represents a core component of this process.

By analyzing published HuR PAR-CLIP data, we further confirmed that HuR binds to the HuB 3′UTR (Fig. S1*C*, GSM741173) (20). Consistent with this, our native RNA-IP assays verified that inflammatory stimulation enhances the association of HuR with HuB mRNA (Fig. 1*F*). We further performed formaldehyde-crosslinked RNA immunoprecipitation (cross-linked RIP) coupled with sonication, which verified that HuR interacts with the 3′UTR of HuB mRNA (Fig. 1*G*). Notably, previous *in vitro* studies have shown that murine HuB binds to multiple sites within its own 3′UTR (21). Accordingly, we also analyzed the binding of HuB to its own mRNA using native RNA-IP and cross-linked RIP assays. Results revealed that inflammatory stimulation promotes the binding of HuB to the 3′UTR of its own mRNA (Figs. 1, *F* and *G* and S1*D*). Furthermore, we designed Cy5-labeled probes containing the ARE motif within the HuB 3′UTR, and RNA electrophoretic mobility shift assays (RNA-EMSA) further validated that both HuR and HuB can directly bind to the HuB 3′UTR (Fig. 1, *H* and *I*). The specificity of the HuR/HuB-RNA interaction was verified by competition assays, in which unlabeled ARE RNAs competed away the protein-RNA complexes in a dose-dependent manner (Fig. 1, *J* and *K*). Moreover, mutating the ARE sequence in the HuB 3′UTR abrogated the binding of both HuR and HuB to the transcript (Fig. S1*E*). Together, these findings unravel a cooperative HuB-HuR autoregulatory network that governs HuB mRNA stability during inflammatory responses.

### Inflammatory stimulation induces HuB binding to the ARE sequences of inflammatory factor mRNAs

**Figure 2:**
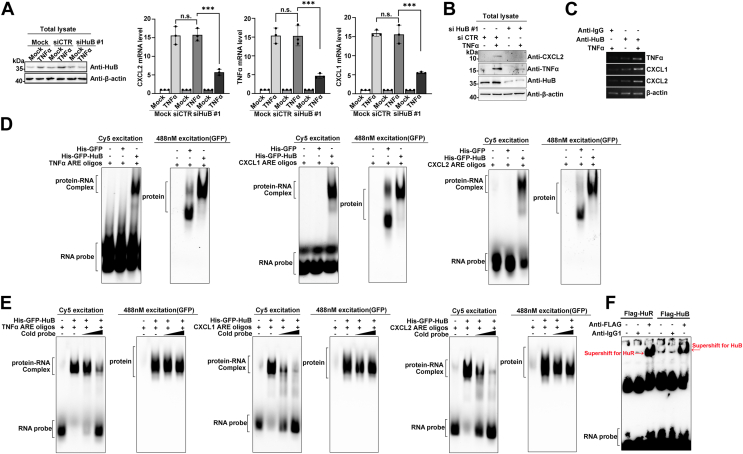
**HuB binds to mRNAs containing AREs and regulates their stability.***A* and *B*, HuB regulates the expression of inflammatory factors. HEK293 cells were transfected with siRNA targeting endogenous HuB or si-CTR. After 48 h of transfection, the cells were treated with TNFα for 1 h. Total cell lysates were extracted, and immunoblotting was performed to detect the expression level of HuB (*left panel of A*). Meanwhile, total RNA was isolated from HEK293 cells after TNFα stimulation. Following reverse transcription, real-time PCR was conducted to measure the mRNA level of inflammatory factors (*right panel of A*). At the same time, immunoblotting was performed to detect the protein expression of inflammatory factors after HuB interference in the cells (*B*). *∗∗∗p* < *0.001*, n.s., no significance. *C*, inflammatory stimulation promotes the binding of HuB to inflammatory factor mRNAs. HEK293 cells were exposed to TNFα. Then, native RNA-IP was performed using control IgG and anti-HuB antibodies. The levels of inflammatory factor mRNAs bound to HuB in the bead-antibody-protein/mRNA complexes were detected by PCR. *D* and *E*, HuB binds to inflammatory factor mRNAs containing ARE sequences. Purified His-GFP and His-GFP-HuB were incubated with Cy5-labeled 3′UTR ARE probes of TNFα, CXCL1, and CXCL2, and protein-probe interactions were detected by RNA-EMSA (*D*). Subsequently, His-GFP-HuB was incubated with the corresponding Cy5-labeled probes in the presence or absence of 10-fold, 20-fold, and 50-fold excess unlabeled RNA for cold probe competition assays (*E*). Cy5-labeled probes were used to detect protein-probe complexes and free probes, while GFP signals were used to verify the presence of the target proteins. *F*, HuR and HuB within the cell bind to the same ARE oligos. HEK293 cells were transfected with Flag-HuB or Flag-HuR plasmids, respectively. Cytoplasmic lysates were extracted, incubated with biotin-labeled tandem ARE repeat probes, and RNA-EMSA assays were performed with IgG or anti-FLAG antibody.

**Fig. S2:**
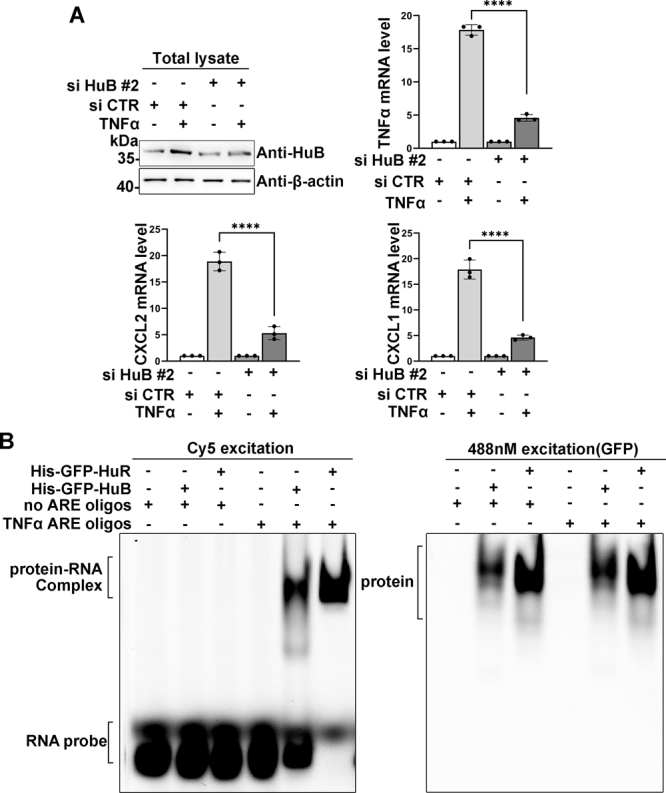


The Hu family represents a canonical family of ARE-binding proteins that bind to mRNAs encoding inflammatory factors, proto-oncogenes, and other targets to regulate mRNA stability (10, 13). Given our observation that HuB is upregulated in TNFα-treated cells, we next investigated whether HuB modulates the expression of inflammatory factors. To address this, we first performed siRNA-mediated HuB knockdown followed by real-time PCR and immunoblotting assays. Results showed that HuB knockdown significantly reduced both the mRNAs and protein expression levels of a panel of inflammatory factors (Figs. 2, *A* and *B* and S2*A*). This was consistent with our previous findings that HuR knockdown inhibits the expression of inflammatory factors (12, 22, 23). Next, we isolated cytoplasmic fractions and performed RNA-IP assays using a HuB-specific antibody. Results demonstrated that HuB binds to the mRNAs of those inflammatory factors (Fig. 2*C*). We then synthesized RNA probes corresponding to the AREs within the 3′UTRs of the inflammatory cytokines TNFα, CXCL1, and CXCL2. *In vitro* RNA-EMSAs were performed using purified recombinant His-GFP-HuB protein. Results demonstrated that HuB directly binds to the ARE regions of these mRNAs (Fig. 2*D*). The specificity of the HuB-RNA interaction was confirmed by competition assays, in which unlabeled ARE RNAs displaced the RNA-protein binding complexes in a dose-dependent manner (Fig. 2*E*). Moreover, the mutation of the TNFα ARE abolished binding by both HuR and HuB (Fig. S2*B*), collectively demonstrating the specificity of HuB binding to the AREs of inflammatory factor mRNAs.

Interestingly, the inflammatory gene mRNAs bound to and regulated by HuB are identical to the target mRNAs of HuR (23). To verify the specific binding of HuR and HuB to the tandem ARE probe, we performed RNA-EMSA using cytoplasmic lysates from cells transfected with Flag-HuR or Flag-HuB under inflammatory stimulation, followed by supershift with IgG or anti-FLAG antibody. Results showed that both proteins formed complexes of nearly identical molecular weight (Fig. 2*F*). Collectively, these findings establish that HuB functions as a critical component of the cytoplasmic ARE-mRNA stabilization network, underscoring its pivotal role in regulating post-transcriptional gene expression during inflammatory stimulation.

### HuB sequesters HuR in the cytoplasm by preventing its shuttling back to the nucleus

**Figure 3:**
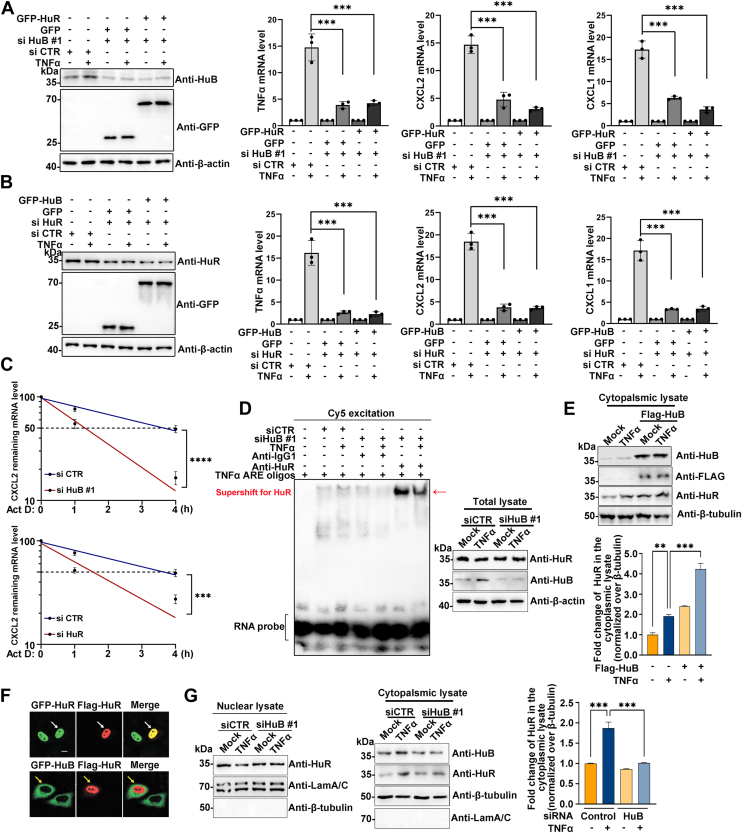
**HuB regulates the subcellular localization of HuR.***A* and *B*, both HuR and HuB are needed for the expression of inflammatory factors. *A*, HEK293 cells were transfected with siRNA targeting endogenous HuB or si-CTR, followed by transfection with GFP or GFP-HuR plasmid. After being mock-treated or exposed to TNFα for 1 h, total cell lysates were extracted, and immunoblotting was performed to detect the expression levels of HuB and GFP/GFP-HuR (*left panel of A*). Meanwhile, total RNA was isolated from HEK293 cells after TNFα stimulation. Following reverse transcription, real-time PCR was conducted to measure the mRNA level of inflammatory factors (*right panel of A*). *B*, HEK293 cells were transfected with siRNA targeting endogenous HuR or si-CTR, followed by transfection with GFP or GFP-HuB plasmid. After being mock-treated or exposed to TNFα for 1 h, total cell lysates were extracted, and immunoblotting was performed to detect the expression levels of HuR and GFP/GFP-HuB (*left panel of B*). Meanwhile, total RNA was isolated from HEK293 cells after TNFα stimulation. Following reverse transcription, real-time PCR was conducted to measure the mRNA level of inflammatory factors (*right panel of B*). *∗∗∗p* < *0.001*. *C*, knockdown of HuB and HuR significantly reduces the half-life of inflammatory factor mRNAs. HEK293 cells were transfected with siRNA targeting endogenous HuB (*upper panel*) or HuR (*lower panel*). After 48 h of transfection, cells were pretreated with TNFα for 20 min, followed by treatment with actinomycin D (Act D, 10 μg/ml). CXCL2 mRNA levels were quantified by real-time PCR at the indicated time points, and mRNA half-lives were calculated.*∗∗∗p* < *0.001*, *∗∗∗∗p* < *0.0001*. *D*, HuB affects the binding of cytoplasmic HuR to ARE-containing target mRNAs. HEK293 cells were transfected with siRNA targeting endogenous HuB or si-CTR. After 48 h of transfection, the cytoplasmic lysate was extracted and incubated with Cy5-labeled 3′UTR ARE probes of TNFα, with IgG or anti-HuR antibody added to the incubation system, followed by RNA-EMSA assay (*left panel*). Meanwhile, the expression levels of HuR and HuB in the cells were detected by immunoblotting (*right panel*). *E*, HuB influences the subcellular localization of HuR. HEK293 cells were transfected with Flag-HuB. After the cells were mock-treated or exposed to TNFα for 1 h, the cytoplasmic lysate was prepared, and the expression of HuR was detected by immunoblotting using an anti-HuR antibody (*upper panel*). The quantitative analysis of the immunoblot results is shown in the *lower panel*. *∗∗p* < *0.01*, *∗∗∗p* < *0.001*. *F*, exogenous overexpression of HuB, but not HuR, promotes cytoplasmic localization of HuR. HEK293 cells were transfected with GFP-HuR or GFP-HuB together with Flag-HuR. The cytoplasm localization of HuR was analyzed by immunofluorescence by anti-FLAG antibody and the GFP fluorescence. *G*, knockdown of HuB reduced nucleocytoplasmic shuttling of HuR. HEK293 cells were transfected with HuB siRNA or si-CTR. After 48 h of transfection, the cells were treated with TNFα for 1 h. Cytoplasmic and nuclear lysates were extracted, and immunoblotting was performed to detect the expression and subcellular localization of HuR (*left and middle panels*). Quantitative analysis of the immunoblot results is shown in the *right panel*. *∗∗∗p* < *0.001*.

**Fig. S3:**
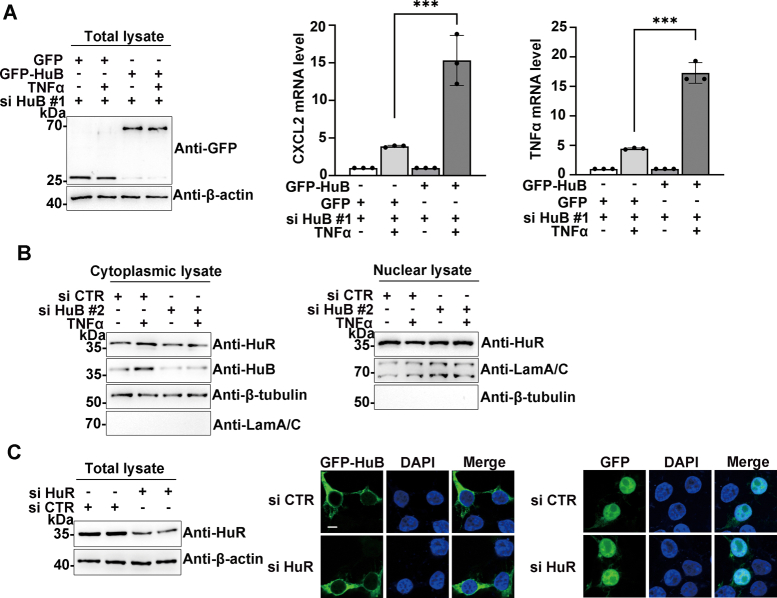


Numerous studies demonstrated the critical role of HuR in regulating the stability of inflammatory factor mRNAs (10, 22, 24, 25); however, our findings demonstrated that HuR reconstitution after HuB knockdown fails to rescue the decreased expression of inflammatory factor mRNAs caused by HuB depletion (Fig. 3*A*), whereas HuB reconstitution restores the expression of these inflammatory factors (Fig. S3*A*). This further supports the functional requirement for high HuB expression in cells under stress conditions. Nucleus-localized HuR is a crucial regulatory protein for the maturation and nuclear export of ARE-containing mRNAs, as well as for maintaining the stability of target mRNAs in the cytoplasm (26, 27, 28). In HuR-knockdown cells, the expression of inflammatory factors was similarly inhibited, even with HuB overexpression (Fig. 3*B*). Knockdown of either HuR or HuB alone significantly reduced the half-lives of inflammatory factor mRNAs (Fig. 3*C*). These results demonstrate that, compared with HuR or HuB alone, HuR and HuB in the cytoplasm play crucial and non-redundant roles in regulating mRNA stability.

Then, we further examined the effect of intracellular HuB on the binding of HuR to mRNA. We successfully knocked down HuB in cells, extracted cytoplasmic lysates, and performed RNA-EMSA using ARE repeat RNA probes. Results revealed that HuB knockdown led to a marked reduction in the abundance of complexes formed by HuR binding to target mRNAs (Fig. 3*D*). Given the presence of excess RNA probes in the assay, which eliminates the possibility of probe limitation, this decrease in HuR-RNA complexes is most likely attributed to HuB regulating the cytoplasmic accumulation of HuR. To test this, we transfected cells with Flag-tagged HuB and subsequently analyzed the subcellular distribution of HuR. As shown in Figure 3*E*, ectopic expression of Flag-HuB led to a marked increase in the cytosolic fraction of HuR. Notably, this effect was further amplified under inflammatory stimulation such as TNFα treatments, indicating that proinflammatory signals synergize with HuB to enhance HuR cytoplasmic accumulation. Consistent with this, co-transfection of GFP-HuB with Flag-HuR induced a clear redistribution of Flag-HuR toward the cytoplasm. In contrast, co-transfection of GFP-HuR with Flag-HuR failed to elicit this relocalization (Fig. 3*F*), confirming that the regulation of HuR cytoplasmic accumulation is specific to HuB. Additionally, siRNA-mediated HuB knockdown completely abrogated TNFα-induced HuR nuclear export (Figs. 3*G* and S3*B*), whereas HuR knockdown had no effect on the cytoplasmic localization of HuB (Fig. S3*C*). Collectively, these results demonstrate that HuB promotes the cytoplasmic accumulation of HuR.

### HuB directly interacts with the HNS domain of HuR *via* its RRM3 domain

**Figure 4:**
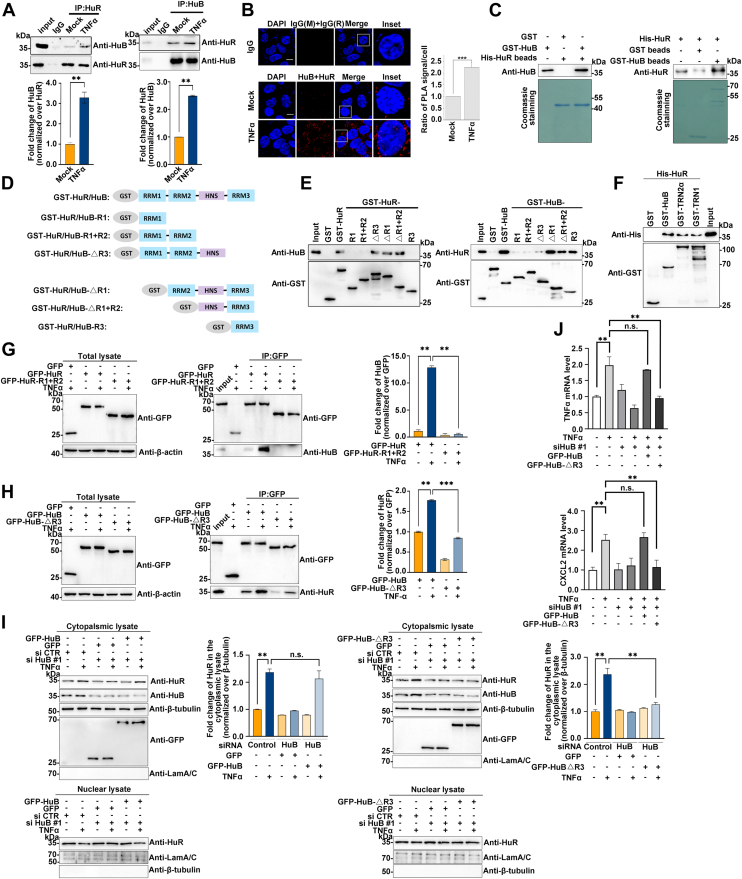
**HuB directly binds to the HNS domain of HuR.***A*, Inflammatory stimulation increases the association of HuB with HuR. HEK 293 cells were either challenged with TNFα for 1 h or not, then immunoprecipitates were prepared using an anti-HuR or HuB antibody and subjected to immunoblotting analysis with the anti-HuB or HuR antibody. *∗∗p* < *0.01*. *B*, PLA was performed to detect the interaction between HuB and HuR. HEK293 cells were mock-treated or exposed to TNFα for 1h, and then subjected to *in vivo* PLA with indicated antibodies.*∗∗∗p* < *0.001*. Scale bar, 10 μm. *C*, HuB directly interacts with HuR. Recombinant GST, GST-HuB, and His-HuR proteins were purified from *E. coli* and subjected to *in vitro* pull-down assays. Bound proteins were detected by immunoblotting using anti-HuB or anti-HuR antibodies. GST and GST-HuB were visualized by Coomassie blue staining as loading controls. *D*, schematic diagram of the truncated domains of GST-HuB and GST-HuR. *E*, the HNS domain of HuR binds to the RRM3 domain of HuB. Equal amounts of His-HuB or His-HuR were incubated with GST and GST-HuR or GST-HuB domains. The bound proteins were analysed by immunoblotting. *F*, HuR preferentially binds to HuB rather than TRN1 and TRN2. Equal amounts of GST-HuB, GST-TRN1, or GST-TRN2α were incubated with His-HuR. The bound proteins were analyzed by immunoblotting. *G* and *H*, the HNS domain of HuR interacts with the RRM3 domain of HuB in cells. Cells were transfected with GFP-HuR or GFP-HuR-RRM1+RRM2 (GFP-HuR-R1+R2) (*left panel of G*), or with GFP-HuB or GFP-HuB-ΔRRM3 (GFP-HuB-ΔR3) (*left panel of H*) plasmid. After being mock-treated or exposed to TNFα for 1 h, immunoprecipitates were prepared using an anti-GFP antibody, and immunoblot analysis was performed with anti-HuB antibody (*middle panel of G*) or anti-HuR antibody (*middle panel of H*) to identify the domains responsible for the interaction between HuR and HuB in cells. The quantitative analysis of the immunoblot results is shown in the *right panel* of *G* or *H*. *∗∗p* < *0.01*, *∗∗∗p* < *0.001*. *I*, tThe HuB-RRM3 domain promotes cytoplasmic localization of HuR. HEK293 cells were transfected with GFP-HuB (*left panel*) or GFP-HuB-ΔRRM3 (GFP-HuB-ΔR3) (*right panel*) plasmid after HuB knockdown. After being mock-treated or exposed to TNFα for 1 h, nuclear and cytoplasmic protein extracts were prepared, and the cytoplasmic localization of HuR was detected by immunoblotting using an anti-HuR antibody. *∗∗p* < *0.01.* n.s., no significance. *J*, the HuB-RRM3 domain is critical for regulating inflammatory factor mRNA stability. HEK293 cells were transfected with GFP-HuB or GFP-HuB-ΔR3 plasmids after HuB knockdown. Cells were pretreated with TNFα for 20 min, followed by treatment with actinomycin D (10 μg/ml) for 4 h with continuous TNFα exposure. TNFα (*upper panel*) and CXCL2 (*lower panel*) mRNA levels were quantified by real-time PCR at the indicated time points, and mRNA half-lives were calculated accordingly. *∗∗p* < *0.01.* n.s., no significance.

**Fig. S4:**
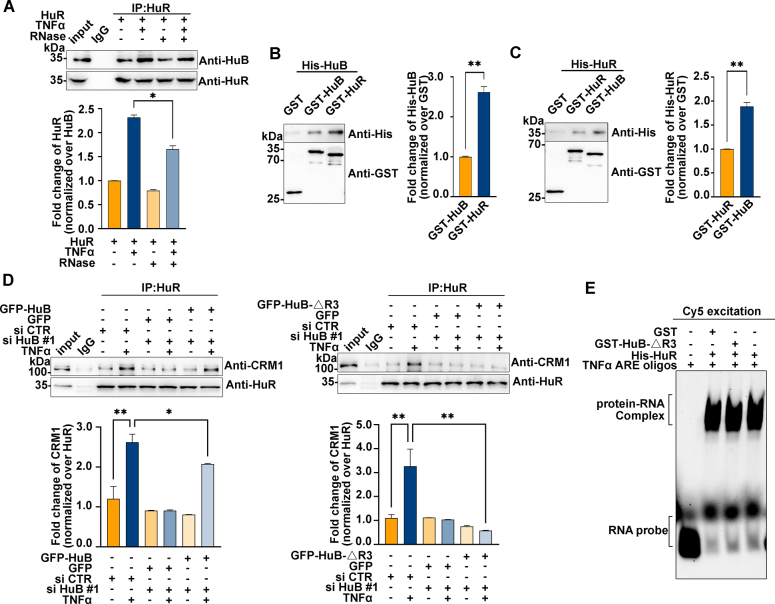


Hu family proteins were reported to form oligomers (homo/hetero) to regulate mRNA stability (29, 30). As HuB binds to the same target mRNAs as HuR and is required for HuR's cytoplasmic localization, we next sought to investigate whether HuB and HuR form a complex in cellular contexts. To address this, cell lysates were subjected to reciprocal Co-IP assays using either anti-HuB or anti-HuR antibody. Immunoblotting analysis confirmed a physical interaction between HuB and HuR under basal conditions, and this interaction was significantly enhanced following TNFα stimulation (Fig. 4*A*). Co-IP assays performed in the presence of RNase revealed that the interaction between HuR and HuB was not RNA-dependent, whereas RNA enhanced their binding (Fig. S4*A*). To further validate this interaction *in situ*, we performed proximity ligation assays (PLA). Cells were treated with or without TNFα, and PLA was conducted using probe pairs specifically recognizing HuB and HuR. Upon TNFα stimulation, we observed a marked increase in the number of PLA signals, which represented close-proximity interactions between HuB and HuR, compared to untreated controls (Fig. 4*B*), providing direct visual evidence of enhanced HuB-HuR complex formation under inflammatory conditions.

We then confirmed a direct physical interaction between HuB and HuR using *in vitro* pull-down assays with purified recombinant proteins, including GST-HuB, GST-HuR, and His-HuR (Fig. 4*C*). Notably, quantitative analysis of these assays revealed that the heterotypic interaction between HuB and HuR was stronger than the homodimeric interactions of either protein (Fig. S4, *B* and *C*), indicating a preferential association between the two family members. All Hu family proteins share a conserved structural architecture, consisting of the three RNA recognition motifs (RRMs) and a flexible hinge region (HNS) connecting RRM2 and RRM3. To map the domains mediating HuB-HuR binding, we constructed a series of truncated GST-tagged HuR and HuB expression plasmids (Fig. 4*D*), respectively, and performed pull-down assays with these recombinant proteins. Our results identified that the HuB-HuR interaction is specifically mediated by the RRM3 domain of HuB and the HNS domain of HuR (Fig. 4*E*), defining the critical structural determinants of their interaction. In cells, importin α/β, transportin 1 (TRN1), and transportin 2 (TRN2) of the karyopherin superfamily mediate HuR nuclear import by interacting with its HNS domain, and HuR exhibited stronger interactions with TRN1 and TRN2 (31, 32, 33). Next, we purified His-HuR, as well as GST-HuB, GST-TRN1, and GST-TRN2α proteins and performed pull-down assays. The results showed that when equal amounts of GST-HuB, GST-TRN1, or GST-TRN2α were incubated with His-HuR, HuB exhibited stronger binding to HuR (Fig. 4*F*). All the above results demonstrate that HuR has a high binding capacity for the RRM3 domain of HuB, and HuR preferentially binds to HuB relative to TRN1 and TRN2, indicating that cytoplasmic HuB has a greater binding capacity for HuR than other import proteins, thereby driving HuR's cytoplasmic retention.

Next, we investigated whether the interaction between HuR and HuB in cells is dependent on the HNS domain of HuR and the RRM3 domain of HuB. Wild-type and truncated plasmids were transfected into cells, and Co-IP assays revealed that deletion of the HuR HNS domain or the HuB RRM3 domain both markedly impaired their direct physical interaction (Fig. 4, *G* and *H*). These findings confirm that HuR and HuB mediate their physical interaction *via* the HuR HNS domain and HuB RRM3 domain, respectively. Following successful HuB knockdown in cells, wild-type and RRM3-deficient HuB constructs were individually transfected. Deletion of the HuB RRM3 domain markedly reduced the interaction between HuR and nuclear export proteins, as well as reduced HuR cytoplasmic accumulation (Figs. S4, *D* and *I*). While deletion of the HuB-RRM3 domain did not impair HuR binding to mRNAs (Fig. S4*E*), the reduced HuR cytoplasmic accumulation induced by HuB-RRM3 domain deletion in cells markedly decreased the half-lives of inflammatory factor mRNAs (Fig. 4*J*). Collectively, HuB interacts with HuR *via* its RRM3 domain and HuR’s HNS domain, which are essential for HuR’s cytoplasmic retention and inflammatory factor mRNA stability.

### The HuB-HuR complex is essential for the expression of inflammatory factors and the inflammatory process

**Figure 5:**
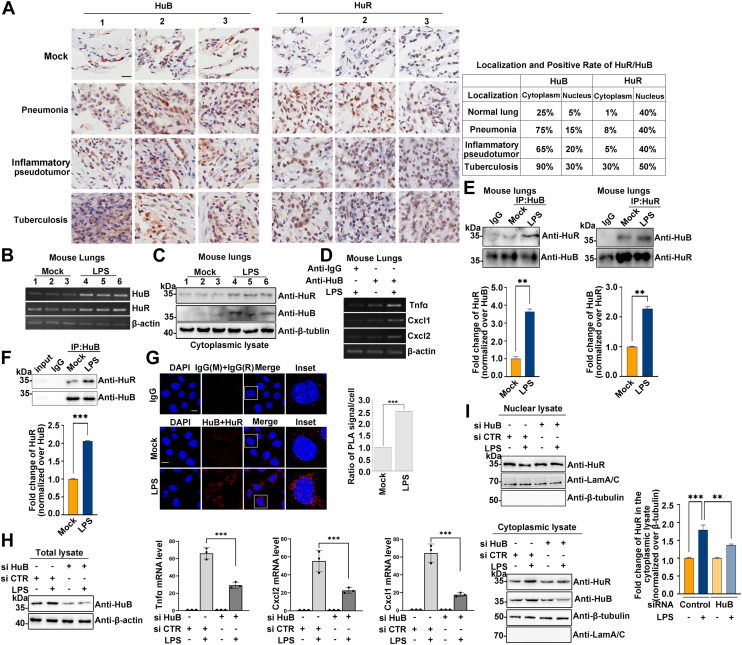
**Inflammatory factor expression and the entire inflammatory process rely on the HuB–HuR complex.***A*, the expression and localization of HuB and HuR in 12 pairs of lung adenocarcinoma and their adjacent non-tumor tissues were examined by IHC. Immunohistochemistry showed the expression of HuR and HuB in 12 patient cases. The expression intensities of HuB and HuR in both the nucleus and cytoplasm were statistically analyzed based on immunostaining results. The evaluation of HuR and HuB localization in cells were performed in a blinded manner by two certified pathologists, with scoring criteria based on staining intensity as described in Experimental procedures. Scale bar, 50 μm. *B* and *C*, inflammatory stimuli upregulate HuB expression *in vitro* and *in vivo*. C57BL/6 mice were intranasally administered LPS (1 mg/kg) for 1 h before lung tissue collection. HuB expression was assessed by PCR (*B*) using lung homogenates and by immunoblotting (*C*) using cytoplasmic extracts from mouse lung tissues. *D*, inflammatory stimulation promotes the binding of HuB to inflammatory factor mRNAs. Mouse lungs were exposed to LPS. Then, native RNA-IP was performed using control IgG and anti-HuB antibodies. The levels of inflammatory factor mRNAs bound to HuB in the bead-antibody-protein/mRNA complexes were detected by RT-PCR. *E*, LPS promotes the interaction between HuB and HuR in mouse lung. Mouse lungs were challenged with LPS (1 mg/kg) for 1h. Then lung homogenates were prepared, immunoprecipitates were prepared using an anti-HuR or HuB antibody and then subjected to immunoblotting analysis with the anti-HuB or HuR antibody. *∗∗p* < *0.01*. *F*, LPS enhances the interaction between HuB and HuR in MLE12 cells. MLE12 cells were treated with or without LPS stimulation for 1 h. Whole-cell lysates were extracted, and immunoprecipitation was performed using an anti-HuB antibody, followed by immunoblotting with an anti-HuR antibody to detect the interaction between HuR and HuB. *∗∗∗p* < *0.001. G*, PLA was performed to detect the interaction between HuB and HuR. MLE12 cells were mock-treated or LPS-exposed for 1 h, and then subjected to *in vivo* PLA with indicated antibodies. *∗∗∗p* < *0.001.* Scale bar, 10 μm. *H*, HuB regulates the expression of inflammatory factor mRNAs. MLE12 cells were transfected with siRNA targeting endogenous HuB or si-CTR and then exposed to LPS for 1h. Total cell lysates were extracted, and immunoblotting was performed to detect the expression of HuB (*left panel*). Meanwhile, total RNA was isolated from MLE12 cells after LPS stimulation. Following reverse transcription, real-time PCR was conducted to measure the mRNA level of inflammatory factors (*right panel*). *∗∗∗p* < *0.001. I*, knockdown of HuB reduced nucleocytoplasmic shuttling of HuR. MLE12 cells were transfected with siRNA targeting endogenous HuB or si-CTR, after being mock-treated or exposed to LPS for 1 h, nuclear and cytoplasmic protein extracts were prepared, and the cytoplasmic localization of HuR was detected by immunoblotting using an anti-HuR antibody (*left panel*). The quantitative analysis of the immunoblot results is shown in the *right panel*. *∗∗p* < *0.01*, *∗∗∗p* < *0.001.*

Emerging studies investigated the role of HuR in cancer development and its association with clinicopathological parameters (34, 35). Notably, HuR mRNA and protein levels exhibit no significant differences between normal and cancer tissues (36); its intracellular localization, however, remains critical (36). According to the prediction results from an online database (www.aipufu.com), HuR protein levels show no significant changes across 14 cancer types, including liver hepatocellular carcinoma (LIHC), lung adenocarcinoma (LUAD), and lung squamous cell carcinoma (LUSC) (Fig. S5*A*). In contrast, HuB expression was upregulated in these tumor tissues compared with normal control tissues (Fig. S5*B*). This suggested a pivotal role for HuB in carcinogenesis. Immunohistochemical analysis of human normal lung tissues, pneumonia tissues, inflammatory pseudotumor tissues, and tuberculosis tissues revealed that inflammatory or neoplastic conditions upregulated HuB expression and predominantly promoted cytoplasmic accumulation of HuR. Meanwhile, HuB expression was relatively low in normal lung tissues and was significantly enhanced under inflammatory or neoplastic conditions (Fig. 5*A*). These findings further suggest that HuB may be the key factor driving the cytoplasmic accumulation of HuR.

To further clarify the importance of HuB for HuR localization regulation, we established a mouse model of lung inflammation. After inducing lung inflammation in mice *via* LPS stimulation, we prepared serial sections from mouse lung tissues and subjected them to HE staining as well as immunostaining for HuR and HuB, respectively. Results showed that LPS significantly upregulated HuB expression and induced cytoplasmic accumulation of HuR in lung tissue cells (Fig. S5*C*). Consistent results were observed in lung tissue homogenates of normal and LPS-stimulated mice: HuB mRNA and protein expression was significantly increased (Fig. 5, *B* and *C*), and LPS enhanced the cytoplasmic accumulation of HuR (Fig. 5*C*). Moreover, HuB was also found to bind to the mRNAs of inflammatory factors (Fig. 5*D*) and interact with HuR (Fig. 5*E*) in mouse lungs. Next, we investigated the impact of HuB on HuR localization and inflammatory factor expression in the mouse alveolar epithelial cell line MLE-12. Co-IP and PLA consistently demonstrated that HuB physically interacted with HuR in MLE-12 cells, and LPS stimulation further enhanced this interaction (Fig. 5, *F* and *G*). HuB knockdown not only inhibited the expression of the inflammatory factors *Cxcl1*, *Cxcl2*, and *Tnfα*, but also suppressed the cytoplasmic accumulation of HuR under inflammatory stimulation (Fig. 5, *H* and *I*). Collectively, these results further establish the critical regulatory role of HuB in promoting the cytoplasmic accumulation of HuR and the expression of inflammatory factors in physiological models, while also highlighting the potential of HuB as a biomarker for inflammatory diseases or tumors.

## Discussion

**Figure 6:**
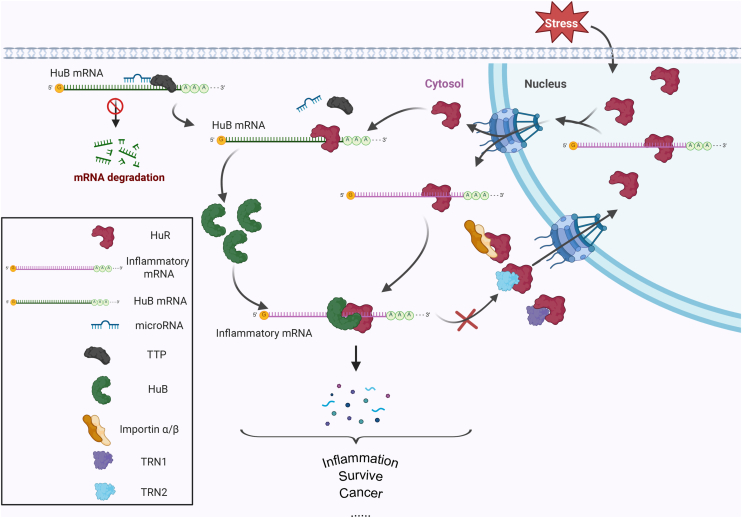
**Schematic Illustration. Schematic diagram of HuR-HuB co-regulation of inflammatory cytokine expression.** Image created with BioRender.com, with permission.

Short-lived mRNAs harboring AREs are critical for cellular stress responses, survival, and immunity. Their half-life directly dictates the abundance of encoded proteins, thereby shaping processes like metabolism, cell death, immune activation, and tumorigenesis (24, 37, 38). The stability of these ARE-containing mRNAs is tightly controlled by Hu family proteins, whose nucleocytoplasmic localization and expression mechanisms remain elusive. Here, we elucidate the regulatory mechanisms underlying stress-induced HuB upregulation and HuR cytoplasmic accumulation and identify a cooperative autoregulatory network between HuR and HuB. Stress stimuli triggers HuR nuclear export, allowing HuR to bind and stabilize HuB mRNA. Upregulated HuB further forms a complex with HuR, promoting HuR cytoplasmic retention and the cooperative regulation of inflammatory factor expression (Fig. 6).

Gene expression regulation is a complex, multi-step, multi-level process. When cells respond to various external stimuli, in addition to the transcriptional regulation of target gene mRNA synthesis, the post-transcriptional control of mRNA stability represents a more sensitive, convenient, and efficient regulatory mechanism evolved in eukaryotic cells (7, 24, 37). Emerging evidence indicates that RBPs often regulate their own expression through autoregulatory feedback mechanisms, such as modulating mRNA stability and/or translational efficiency by binding to their own transcripts (39, 40, 41). For example, HuR autoregulates its expression by influencing alternative polyadenylation site usage, thereby maintaining intracellular homeostasis (19). In the present study, we demonstrate that inflammatory stimulation upregulates both HuB mRNA and protein expression. Mechanistically, inflammatory signals promote the cytoplasmic translocation of HuR, which in turn stabilizes HuB mRNA, and the accumulated HuB protein further enhances this stabilization by binding to its own transcript and cooperating with HuR, thereby forming a positive feedback loop (Fig. 1). These findings broaden the traditional view of autoregulation within the Hu family as a self-contained mechanism and reveal a previously unrecognized interdependent regulatory network.

Interestingly, recent studies have identified that ZFP36L2, a member of the TTP family, binds to the ARE element in the 3′UTR of HuB and regulates HuB decay in the mouse spleen (42). Based on our experimental results (Fig. 1), we propose a model in which, under normal cellular conditions, intracellular degradative ARE-binding proteins such as ZFP36L2 bind to HuB mRNA to maintain its low-level expression; once cells are exposed to external stimuli including inflammatory stimulation and tissue injury stimuli, HuR translocates from the nucleus to the cytoplasm, competes with other ARE-binding proteins or microRNAs for binding sites, stabilizes HuB mRNA, and consequently promotes HuB protein expression. This cooperative autoregulatory mechanism ensures the rapid and sustained upregulation of HuB, serving as a critical adaptive mechanism for cells to mount an effective inflammatory response (Fig. 6). However, how HuR competes with other ARE-binding proteins or microRNAs for binding to HuB mRNA, as well as the underlying molecular mechanisms, remains to be further explored in future studies. Furthermore, although numerous studies showed that the expression of Hu family members is largely regulated at the post-transcriptional level (19, 39, 40, 41, 42), HuR has been shown to act as a direct transcriptional target of NF-κB in gastric cancer cells, and HuR’s activation in gastric cancer cell lines has been demonstrated to rely on the phosphatidylinositol 3-kinase/AKT (PI3K/AKT) signaling pathway (43). Whether the NF-κB signaling pathway can directly affect HuB mRNA levels or indirectly modulate HuB expression by regulating other proteins under inflammatory stimulation also warrants further investigation in future studies.

HuR, predominantly localized in the nucleus, regulates mRNA maturation and forms complexes with mRNA for cotransport into the cytoplasm (26, 44). In the cytoplasm, it stabilizes target mRNAs by inhibiting the binding of other RBPs or microRNAs (13, 26). Our study demonstrates that HuB, highly expressed under inflammatory stimuli, binds to the same ARE-containing mRNAs as HuR but is not a compensatory protein for HuR (Figs. 2 and 3). Silencing either HuR or HuB individually in cells significantly reduces the expression of inflammatory factors, indicating that the HuR-HuB complex is indispensable for inflammatory factor expression and highlighting their non-redundant, synergistic roles in Hu family-mediated post-transcriptional regulation of inflammation (Fig. 3).

To date, fully resolved crystal structures of full-length HuR and HuB proteins remain unavailable, and computationally predicted interactive amino acid residues between the two proteins exhibit low confidence. Our results demonstrate that HuB directly binds to the HNS domain of HuR through its RRM3 domain (Fig. 4). Although crystal structures of these two domains, namely the RRM3 domain of HuB and the HNS domain of HuR, are still unsolved, their direct interaction has been unambiguously verified by our experimental data. These findings lay a solid foundation for future identification of the minimal core amino acid sequence mediating HuR-HuB interaction. As the HNS domain of HuR regulates its nucleocytoplasmic shuttling through the embedded nuclear localization signal (NLS) and nuclear export signal (NES) (45, 46, 47), we propose that HuB, which is highly expressed in the cytoplasm, binds to the HNS domain of HuR to mask HuR’s NLS, blocking interactions between HuR and TRN1, TRN2, as well as importin α/β and thereby retaining HuR in the cytoplasm. Although the potential contributions of additional cofactors or inflammation-specific modifications remain to be elucidated, this study identifies HuB as an inflammation-induced "gatekeeper" that modulates HuR’s cytoplasmic retention and consequently influences the magnitude of the post-transcriptional inflammatory program.

Although HuR has been proposed as a therapeutic target (25, 48), its total expression level exhibits no significant changes under inflammatory stimuli or in various tumors; instead, it accumulates in the cytoplasm (49, 50, 51, 52). Our study demonstrates that HuB expression is significantly upregulated in pneumonia, inflammatory pseudotumor, and tuberculosis tissues, as well as in a mouse model of LPS-induced lung inflammation and is positively correlated with cytoplasmic accumulation of HuR. Knockdown of HuB markedly inhibits HuR cytoplasmic localization and inflammatory factor expression (Fig. 5). These findings suggest the potential of HuB as a diagnostic biomarker for inflammation and cancer and highlight the feasibility of treating inflammatory diseases or tumors by targeting HuB expression or the HuB-HuR interaction.

In conclusion, we elucidate the molecular underpinnings and functional impact of HuB upregulation evoked by inflammatory stimuli. We demonstrate that inflammatory cues trigger the nuclear export of HuR, which post-transcriptionally boosts HuB expression by stabilizing HuB mRNA. Newly induced HuB in turn sequesters HuR in the cytoplasm, assembling an mRNA-stabilizing complex that prevents HuR nuclear re-entry and thereby amplifies the expression of pivotal inflammatory mediators. These findings reveal a self-reinforcing regulatory circuit among Hu family proteins that tunes the inflammatory response and highlight this axis as a candidate therapeutic target in inflammation-driven pathologies, including cancer.

## Experimental procedure

### Antibodies and reagents

The goat polyclonal antibody HuB (1:3000, 14008-1-AP), the CRM1 monoclonal antibody (1: 4000, 66763-1-Ig), and the monoclonal antibody against Lamin A/C (1:2000, 10298-1-AP) were purchased from Proteintech (Wuhan, China). The mouse monoclonal antibody against TNFα (1:1000, 17590-1-AP) was purchased from Proteintech, and the rabbit polyclonal antibody against CXCL2 (1:1000, ab317569) was purchased from Abcam. The monoclonal antibodies against HuR (1:3000, 3A2, sc-5261) was purchased from Santa Cruz Biotechnology (Santa Cruz). The following mouse monoclonal antibodies were purchased from TRANS: anti-β-tubulin (1:8000, HC101), anti-GFP (1:8000, HT801), anti-GST (1:8000, HT601), anti-His (1:8000, HT501), and anti-β-actin (1:8000, HC201). A monoclonal antibody against FLAG (1:8000, F1804) was purchased from Sigma-Aldrich.

The dose of recombinant human TNFα (Peprotech, 300–01A) was 10 ng/ml. Transcription inhibitor actinomycin D (Act D, Sigma, A1410, 10 μg/ml) and lipopolysaccharide (LPS, Sigma, L2630, 500 ng/ml) were added directly to the culture medium.

### Constructs

Plasmid GST-HuB was kindly provided by Dr Hua Lou (Department of Genetics, Case Comprehensive Cancer Center, and Center for RNA Molecular Biology, School of Medicine, Case Western Reserve University, and Cleveland, OH 44106). The domain mutations GST-HuB-RRM1, GST-HuB-RRM2, GST-HuB-HNS, GST-HuB-ΔRRM1, GST-HuB-ΔRRM1+RRM2 and GST-HuB-RRM3 were developed from GST-HuB. To construct GFP-HuB and Flag-HuB plasmids, the full-length of HuB was cloned into the vector pGFP-C1, pCMV-N-FLAG and pET30a. GFP-HuB-ΔRRM3 was developed from GFP-HuB. Plasmids GST and GST-HuR were kindly provided by Dr Myriam Gorospe (Laboratory of Cellular and Molecular Biology; National Institute on Aging, National Institutes of Health). The domain mutations GST-HuR-RRM1, GST-HuR-RRM2, GST-HuR-HNS, GST-HuR-ΔRRM1, GST-HuR-ΔRRM1+RRM2 and GST-HuR-RRM3 were developed from GST-HuR. Plasmid GFP-HuR was provided by Dr Imed-Eddine Gallouzi (Department of Biochemistry, Division of Critical Care, McGill University Health Center, McGill University, Montreal, Quebec H3G 146). GFP-HuR-RRM1+RRM2 was developed by GFP-HuR. To construct Flag-HuR and His-HuR plasmids, the full-length of HuR was cloned into the vector pCMV-N-FLAG and pET30a. GST-TRN1 and GST-TRN2α were kindly provided by Dr Joan A Steitz (Department of Molecular Biophysics and Biochemistry, Howard Hughes Medical Institute, Yale University School of Medicine). To construct His-GFP-HuR or His-GFP-HuB plasmid, the full-length of HuR or HuB was cloned into the vector pHis-GFP.

### Cell culture and treatment

HEK293 and MLE12 cells were cultured in DMEM (Invitrogen) supplemented with 10% (v/v) fetal bovine serum (FBS). Lipofectamine 2000 (Invitrogen) was used to transfect the cells with plasmid DNA, as recommended by the manufacturer. Small interfering RNAs (siRNAs) targeting human HuR (5′-UGCCGUCACCAAUGUGAAAGU-3′), human HuB (#1: 5′-UUAUUGUUUUGGUUUGAAGUC-3′, #2: 5′-GACAGAGUACUGCAGGUCU-3′), mice HuB (5′-CUUCAAACCAAAACAAUAAAA-3′), and siControl (siCTR, 5′-UUCUCCGAACGUGUCACGUUU-3′) were used at a concentration of 100 nM. The cells were transfected with RNA oligos using Lipofectamine 3000 (Invitrogen), following the manufacturer's instructions.

### RNA isolation, cDNA synthesis, and real-time PCR

Total RNA was extracted using TRIzon Reagent (CW0580S, CWBIO, China), and 1 μg of purified RNA from each sample was transcribed to cDNA by the PrimeScriptRT reagent Kit plus gDNA Eraser (RR047A, Takara) according to the manufacturer’s instruction. Real-time PCR was performed on the QuantStudio 3 Real-time PCR Instrument (Applied Biosystems) with a TB Green Premix Ex Taq (Tli RNaseH Plus) regent (RR420A, Takara). The mRNA expression levels of the target genes were normalized to the expression levels of the β-actin gene. The oligonucleotide primer pairs used in this study are shown in Table S1 and were synthesized by Comate Bioscience (Changchun, China).

Quantitative real-time PCR was performed to detect mRNA levels, and relative mRNA expression was calculated as fold change using the ΔΔCt method.

### Cell fractionation

Whole-cell lysis, as well as the isolation of the cytosolic and nuclear fractions, was performed as previously described (24). Briefly, whole-cell lysates were prepared in radioimmunoprecipitation assay (RIPA) buffer on ice for 30 min. The lysates were then centrifuged, and the resulting supernatant was the whole cell lysis buffer (WEs). The cytoplasmic and nuclear fractions were prepared using the CelLytic NuCLEAR Extraction Kit (Sigma). The cells were lysed with cytosolic lysis buffer for 20 min. The lysates were then centrifuged at 110,000*g* for 1 min at 4 °C, and the resulting supernatant was collected as the cytosolic extract (CEs). The pellets were washed twice with cytosolic lysis buffer and lysed with extraction buffer. The nuclear lysates were clarified by centrifugation (210,000*g* for 5 min at 4 °C) and the resulting supernatant was collected as the nuclear extract (NEs).

### Immunoblotting and immunoprecipitation

Cells were cultured and stimulated as described above, after which they were lysed. Then, the proteins in each sample were separated using SDS-PAGE. The proteins were then transferred to nitrocellulose (NC) membranes, which were washed with TBST (20 mM Tris base, 500 mM NaCl, 0.1% Tween-20, pH 7.5) and blocked with 5% non-fat dry milk. Blot signals were detected using an ECL chemiluminescent detection system after incubation overnight with a primary antibody and a horseradish peroxidase-conjugated secondary antibody.

For co-immunoprecipitation analysis, CE or WE were incubated with antibodies recognising HuB or HuR, and then with protein G/A agarose/salmon. IgG (Santa Cruz) was used for controlling IP reactions. Protein–protein interactions were studied by immunoblotting analysis of IP samples.

The relative band intensities were quantified by densitometry using the ImageJ software (1.41V, US National Institutes of Health).

### Immunofluorescence microscopy

Cells were first fixed with 10% (v/v) formaldehyde. After permeabilized with 0.5% (v/v) Triton X-100, cells blocked with 2% (w/v) bovine serum albumin and incubated with primary antibodies recognizing FLAG (1:200). Secondary antibodies were used to detect primary antibody-antigen complexes with different color combinations as needed. The nuclei of the cells were stained with DAPI for 5 min. Images were acquired using a confocal microscope (LSM880, ZEISS, Germany).

### Duolink (proximity ligation assay, PLA)

The proximity ligation assay (PLA) (Dolin In Situ Detection Reagents Red, DUO92008, sigma) was performed following the manufacturer’s instructions. Cells were growing on slides, fixed with paraformaldehyde 4% and blocked for 1 h, then incubated with the primary antibodies against HuR and HuB overnight at 4 °C. IgG was used as a negative control. Each dot corresponds to a close interaction. A pair of oligonucleotide-labeled secondary antibodies (PLA probes) binds to the primary antibodies and generates a signal only when the two probes are in proximity.

### Stability of mRNA

To measure the stability of HuB mRNA, a classical approach is applied. HEK293 cells were exposed to TNFα for 20 min, then transcription inhibitor Act D was added to the medium with or without the maintenance of TNFα for 0, 2, 4 and 6 h or 0, 1, and 4 h. The level of mRNA was measured by real-time PCR assay.

### Recombinant protein purification and pull-down assay

GST/His/His-GFP and GST/His/His-GFP-fused proteins were expressed in *E. coli* strain BL21. The induction was performed by adding 1 mM isopropyl- β-D-thiogalactopyranoside to an OD 1.0 culture at 37 °C for ∼3 to 4 h. Pelleted cells were re-suspended in lysis buffer (250 mM NaCl, 50 mM HEPES PH7.5, 1 mM DTT, protease inhibitor). After sonication, lysates were centrifuged at 30,000*g* at 4 °C for 30 min. The supernatant (with 0.5% Nonidet P-40) was collected and incubated with balanced and reduced GST-tag Purification Resin (P2253, Beyotime Biotechnology) or His-tag Purification Resin (P2218, Beyotime Biotechnology) on a rotator at 4 °C overnight. Beads were washed and eluted, and the puried recombinant proteins were confirmed by immunoblotting.

For pull-down experiments, GST and GST-fused proteins immobilized on Glutathione Sepharose 4B were incubated with His-fused proteins at 4 °C for 3 h. After three washes with Nonidet P-40 lysis buffer, the bound proteins were analyzed by immunoblotting.

### RNA-EMSA

To perform RNA EMSA and supershift analyses, a Chemiluminescent RNA EMSA Kit (20158, Thermo Fisher Scientific) was used. Briefly, GST or GST-HuB proteins were purified and eluted in 100 μl buffer (50 mM Tris–HCl (pH > 8.0), 100 mM KCl and 40 mM glutathione). GST, GST-HuB proteins or cells cytoplasm lysis buffer were dissolved in the EMSA interaction buffer (1 mM MgCl2, 20 mM KCl, 5% glycerol, 10 ng tRNA) and incubated with 5 nM of 5′ biotin-labeled RNA oligos for 30 min at room temperature. For supershift assays, 0.4 μg of specific antibodies or IgG were added to the mixture after 15 min of incubation at room temperature. The reaction mix was then loaded onto a 6% acrylamide native gel. RNA oligonucleotide probes used in this study were purchased from Sangon Biotech, as listed in Table S2.

### RNA immunoprecipitation (RNA-IP) and formaldehyde-crosslinked RNA immunoprecipitation (cross-linked RIP)

For native RNA immunoprecipitation (RNA-IP) assays, HEK293 cells were lysed in cytoplasmic lysis buffer (20 mM Tris-HCl, pH 7.5, 100 mM KCl, 5 mM MgCl_2_, and 0.3% NP-40) for 5 min and then centrifuged at 10,000*g* for 10 min at 4 °C. The supernatants were collected as cytoplasmic fractions and used for native RNA-IP. The cytoplasmic fractions were incubated with protein G Sepharose beads coated with anti-HuR/HuB antibody or an equal amount of IgG (4 μg) for 3 h. After the beads were washed with NT2 buffer (50 mM Tris-HCl, pH 7.4, 150 mM NaCl, 1 mM MgCl_2_, and 0.05% Nonidet P-40), one-half of the bead-antibody-protein/mRNA complexes from each sample were analyzed by immunoblotting, and the other half was treated with 20 units of RNase-free DNase I (15 min at 37 °C) and 0.5 mg/ml proteinase K (15 min at 55 °C) to remove DNA and protein, respectively. RNA was isolated by phenol-chloroform extraction and subjected to real-time PCR array analysis.

For formaldehyde-crosslinked RNA immunoprecipitation (cross-linked RIP) assays, mock- or TNFα-treated cells were crosslinked in 1% formaldehyde for 10 min at room temperature and sonicated to an average RNA size of 300 bp in lysis buffer (50 mM Tris-HCl, pH 8.0, 10 mM EDTA, 1% SDS, and 1 × protease inhibitor cocktail) using a Qsonica sonicator. The supernatants were diluted with dilution buffer (15 mM Tris-HCl, pH 8.0, 1 mM EDTA, 150 mM NaCl, 1% Triton X-100, 0.01% SDS, and protease inhibitors) and incubated with appropriate antibodies overnight at 4 °C. After the complexes were captured with Protein-G magnetic beads (Millipore), the beads were washed twice in buffer I (20 mM Tris-HCl, pH 8.0, 150 mM NaCl, 1 mM EDTA, 1% Triton X-100, and 0.1% SDS), once in buffer II (same as buffer I but containing 500 mM NaCl), and finally with 1 × Tris-EDTA (TE, pH 8.0) buffer. RNA was eluted from the beads with elution buffer (1.0% SDS and 100 mM NaHCO_3_), and cross-linking was reversed by incubation at 65 °C for 2 h. RNA was isolated by acidic phenol–chloroform extraction followed by ethanol precipitation. One microgram of purified RNA from each sample was reverse-transcribed to cDNA. Primers used for PCR are shown in the Table S1.

### Human tissue microarray and immunohistochemistry (IHC)

The lung adenocarcinoma tissue chip (HLugI040PT01, Outdo Biotech, Shanghai, China) incorporates 40 pairs of surgical lung adenocarcinomas and adjacent non-tumour specimens. All cases were diagnosed and staged according to the seventh edition International Union Against Cancer/American Joint Committee on Cancer TNM classification. This protocol was approved by the Ethics Review Committee of the Second Hospital of Shanghai Outdo Biotech Company and the study was performed according to ethical and safe research practices of human subjects or tissues. Informed consent was obtained from all patients.We selected three cases of normal human lung tissue, three cases of pneumonia, three cases of inflammatory pseudotumor of the lung, and three cases of pulmonary tuberculosis for immunohistochemical staining.

The expression and location of HuB or HuR in each tissue were examined by IHC. Briefly, tissue sections (3 μm) were deparaffinized, rehydrated, incubated with 3% H2O2 in methanol and subjected to antigen retrieval by EDTA buffer. The sections were blocked with 5% bovine serum albumin (BSA), probed with anti-HuB(1:600), or anti-HuR(1:600) antibody at 4 °C overnight. The sections were reacted with biotinylated secondary antibodies and detected using the Streptavidin-Peroxidase IHC assay kit and DAB (ZSGB-bio). Immunostaining was evaluated in a blinded manner by two experienced and certified pathologists. The evaluation result was calculated as the product of the staining intensity of HuR or HuB protein in the cytoplasm or nucleus and the percentage of positive cells, and the final result was presented as a percentage.

### Mouse work

Six-to eight-week-old female C57BL/6 mice (20–25*g*) were purchased from Jilin University. Mice were housed in a specific pathogen-free facility at NENU and allowed unlimited access to sterilized feed and water. They were maintained at 23 ± 1 °C and kept under a 12-h light/dark cycle. All experiments were conducted in accordance with the Chinese Council on Animal Care Guidelines. Mice were challenged with LPS (1 mg/kg) *via* intranasal administration, and then mice's lungs were harvested, HE and immunohistochemical (IHC) staining analyses were performed. Concurrently, lung homogenates were prepared, and total RNA was extracted from lung tissues. Following reverse transcription, the mRNA levels of HuB and HuR were determined. Additionally, cytoplasmic lysates were isolated to detect the protein levels of HuB and HuR in the cytoplasm.

### Statistical analysis

All experiments were performed at least three times for each determination. Data were expressed as means ± standard deviations and analyzed by one-way analysis of variance. The level of significance was accepted at *∗∗p* < *0.01*, *∗∗∗p* < *0.001*, *∗∗∗∗p* < *0*.*0001.*

## Data availability

Raw data for blots and source data for graphs can be found in Supplemental File Sets (uncropped gel images and the source data behind the graphs in the paper). All data generated or analyzed during this study are available from the lead contact on reasonable request.

## Supporting information

This article contains supporting information.

## Conflict of interest

The authors declare that they have no conflicts of interest with the contents of this article.

## Figures and Tables

**Fig. S5:**