# Supplementary material for: HuR-HuB autoregulatory network governs inflammatory factors expression
Source: J Biol Chem. 2026 May 20;302(7):113175. doi: 10.1016/j.jbc.2026.113175 (PMC13276331; doi:10.1016/j.jbc.2026.113175)
Supplement: Supplement Table [file mmc1.pdf]

Supplement table1

Detected primers

Human HuB	Forward: 5’-TGGACGCATTATTACTTCT-3’<br>Reverse: 5’-CTTGGGTTATTAGCAAACT-3’
Human β-actin	Forward: 5’-CTCCATCCTGGCCTCGCTGT-3’<br>Reverse: 5’-GCTGTCACCTTCACCGTTCC-3’
Human HuB 5’UTR	Forward: 5’-GGGTGAGGAGGGCGGTTG-3’<br>Reverse: 5’-CCTGCTGCTGTGACTCGAA-3’
Human HuB CDS	Forward: 5’-TGGACGCATTATTACTTCT-3’<br>Reverse: 5’-CTTGGGTTATTAGCAAACT-3’
Human HuB 3’UTR	Forward: 5’-TCAGTTGGTTGTGTCTTT-3’<br>Reverse: 5’-ATAATGTAGCCCCCCCTT-3’
Human CXCL2	Forward: 5’-CAAACCGAAGTCATAGCC-3’<br>Reverse: 5’-GAACAGCCACCAATAAGC-3’
Human CXCL1	Forward:5’-TCTCTCTTTCCTCTTCTGTTCCTA-3’<br>Reverse: 5’-CATCCCCCATAGTTAAGAAAATCATC-3’
Human TNFα	Forward: 5’-CAGCTTGAGGGTTTGCTAC-3’<br>Reverse: 5’-TGCACTTTGGAGTGATCGG-3’
Murine HuR	Forward: 5’-AACTGCCTCACCTACTGG-3’<br>Reverse: 5’-AGTGGGAGGGTCCTTATT-3’
Murine HuB	Forward: 5’-ATGGATACCGCCTGGGAGA-3’<br>Reverse: 5’-GCTAAGTAGTCATTTATCCC-3’
Murine *β-actin*	Forward: 5’-AACAGTCCGCCTAGAAGCAC-3’<br>Reverse: 5’-CGATGACATCCGTAAAGACC-3’
Murine *Cxcl2*	Forward: 5’-CACTCTCAAGGGCGGTCAAA-3’<br>Reverse: 5’-TGGTTCTTCCGTTGAGGGAC-3’
Murine *Cxcl1*	Forward: 5’-TGGCTGGGATTCACCTCAAG-3’<br>Reverse: 5’-CCGTTACTTGGGGACACCTT-3’
Murine *Tnfα*	Forward: 5’-AGACCCTCACACTCAGATCA-3’<br>Reverse: 5’-TCTTTGAGATCCATGCCGTTG-3’

Supplement table2

RNA oligo probes

Biotin-labeled tandem ARE repeat RNA oligo	5'-Biotin-AUUUAUUUAUUUAUUUAUUUAUUUA-3'
Cy5-labeled TNFα ARE oligo:	5'-Cy5-AUUAUUUAUUAUUUAUUUAUUAUUUA-3'
Cy5-labeled TNFα no ARE oligo:	5'-Cy5-AAGAUGUAUCAUGUAUCUAUGAUCUA-3'
TNFα ARE oligo (cold probe):	5'-AUUAUUUAUUAUUUAUUUAUUAUUUA-3'
Cy5-labeled CXCL1 ARE oligo:	5'-Cy5-UUCUAUUUAUUUAUUUAUUCAUU-3'
CXCL1 ARE oligo (cold probe):	5'-UUCUAUUUAUUUAUUUAUUCAUU-3'
Cy5-labeled CXCL2 ARE oligo:	5'-Cy5-CUAUUUAUUUAUUUAUUUAUUUAUUUGU-3'
CXCL2 ARE oligo (cold probe):	5'-CUAUUUAUUUAUUUAUUUAUUUAUUUGU-3'
Cy5-labeled HuB ARE oligo:	5'-Cy5-AAACAGUAUUUAUUUUGUAAUUAUGA-3'
HuB ARE oligo (cold probe):	5'-AAACAGUAUUUAUUUUGUAAUUAUGA-3'
HuB no ARE oligo :	5'-Cy5-AAACAGUAUGGAUCCUGUAAUGAUGA-3'
