# Supplementary material for: HuR-HuB autoregulatory network governs inflammatory factors expression
Source: J Biol Chem. 2026 May 20;302(7):113175. doi: 10.1016/j.jbc.2026.113175 (PMC13276331; doi:10.1016/j.jbc.2026.113175)
Supplement: Supplementary Material [file mmc2.doc]

**Supplementary Figure Legends**

**Fig. S1 HuR binds to the 3'UTR of HuB mRNA and mediates HuB upregulation**

**(A)** Time dynamics of HuR shuttling to the cytoplasm. HEK293 cells were treated with TNFα for 0, 0.5, 1, 2, 3 and 4 h. Cytoplasmic and nuclear lysates were extracted, and immunoblotting was performed to detect the expression and subcellular localization of HuR (left and middle panel). Quantitative analysis of the immunoblot results is shown in the right panel. ***p* < *0.01.*

**(B)** Restoration of HuR expression in cells with efficient HuR knockdown can rescue the expression of HuB. HEK293 cells were transfected with GFP or GFP-HuR plasmids after endogenous HuR knockdown. After being mock-treated or exposed to TNFα for 1 h, total cell lysates were extracted, and immunoblotting was performed to detect the expression of HuB and HuR.

**(C)** HuR interacts with HuB mRNA 3'UTR. Published photoactivatable ribonucleoside-enhanced crosslinking and immunoprecipitation (PAR-CLIP) data demonstrate that HuR specifically binds to the 3'UTR region of HuB mRNA. The PAR-CLIP peaks were mapped to the HuB 3'UTR sequence, with significant enrichment compared to background controls.

**(D)** HuB binding to the 3'UTR of its own mRNA. HEK293 cells were exposed to TNFα. Then cross-linked RIP assays were performed using control IgG or anti-HuB antibodies.The bead-antibody-protein/mRNA complexes were subjected to PCR to detect the specific binding sites of HuB.

**(E, F)** HuR and HuB bind specifically to the ARE sequences within the 3'UTR of HuB. Purified His-GFP-HuR (E) and His-GFP-HuB (F) proteins were incubated with Cy5-labeled HuB-ARE probe and HuB probe with mutated non-functional ARE (no ARE oligonucleotides). Protein-probe interactions were detected by RNA-EMSA. Cy5-labeled probes were applied to visualize protein-probe complexes and free probes, whereas GFP fluorescence signals were detected to confirm the presence of target proteins.

**Fig. S2 HuB interacts with ARE-containing mRNAs and modulates their stability**

**(A)** Knockdown of HuB reduces the expression of intracellular inflammatory cytokines. HEK293 cells were transfected with siRNA targeting endogenous HuB or si-control (si-CTR). After 48 h of transfection, the cells were treated with TNFα for 1 h. Total cell lysates were extracted, and immunoblotting was performed to detect the expression level of HuB. Meanwhile, total RNA was isolated from HEK293 cells after TNFα stimulation. Following reverse transcription, real-time PCR was conducted to measure the mRNA level of inflammatory factors. *****p* < *0.0001*.

**(B)** HuR and HuB bind specifically to ARE sequences. Purified His‑GFP, His‑GFP‑HuR, and His‑GFP‑HuB were incubated with Cy5‑labeled TNFα-ARE probe and TNFα probe with mutated non-functional ARE (no ARE oligos). Protein‑probe interactions were determined by RNA‑EMSA. Cy5‑labeled probes were used to detect protein‑probe complexes and free probes, while GFP signals were used to verify the presence of the target proteins.

**Fig. S3 HuB modulates the expression of inflammatory cytokines and the subcellular localization of HuR**

**(A)** HuB promotes the expression of inflammatory factors. HEK293 cells were transfected with siRNA targeting HuB, followed by transfection with GFP or GFP‑HuB plasmid. After being mock-treated or exposed to TNFα for 1 h, the expression levels of inflammatory factors were detected by real‑time quantitative PCR. ****p* < *0.001*.

**(B)** Knockdown of HuB reduces the shuttling of HuR. HEK293 cells were transfected with HuB siRNA or si-CTR, after 48 h of transfection, the cells were treated with TNFα for 1 h. Cytoplasmic and nuclear lysates were extracted, and immunoblotting was performed to detect the expression and subcellular localization of HuR.

**(C)** HuR does not affect the intracellular localization of HuB. HEK293 cells were transfected with siRNA targeting HuR, followed by transfection with GFP or GFP‑HuB plasmid. Immunofluorescence (IF) was performed to examine whether HuR knockdown affects the intracellular localization of HuB. Scale bar, 10 μm.

**Fig. S4 The interaction between HuR and HuB is stronger than their respective homodimerization**

**(A)** RNA enhances the interaction between HuR and HuB. HEK293 cells were treated with TNFα for 1 h, and cytoplasmic lysates were collected. After RNase treatment, Co-IP assays were performed using HuR antibody and subjected to immunoblotting analysis with the anti-HuB or HuR antibody (upper panel). The quantitative analysis of the immunoblot results is shown in the lower panel. **p* < *0.05*.

**(B, C)** The interaction of HuB and HuR is stronger than their respective homodimerization. GST, GST-HuR, GST-HuB and His-HuB (B), His-HuR (C) were purified from bacteria and incubated with each other. The bound proteins were analyzed by immunoblotting with the anti-His antibody. ***p* < *0.01*.

**(D)** The HuB-RRM3 domain promotes cytoplasmic localization of HuR. HEK293 cells were transfected with GFP-HuB or GFP-HuB-ΔR3 plasmid after HuB knockdown. Following mock treatment or TNFα stimulation for 1 h, nuclear protein extracts were prepared. Immunoprecipitates were prepared using an anti-HuR antibody and analyzed by immunoblotting with an anti-CRM1 antibody to identify the domains responsible for the interaction between HuR and CRM1 in cells (upper panel of D). The quantitative analysis of the immunoblot results is shown in the lower panel of D. **p* < *0.05*, ***p* < *0.01*.

**(E)** The HuB-RRM3 domain does not affect the binding of HuR to its target RNA. Purified His-HuR alone, or together with GST or GST-HuB-ΔR3 protein, was incubated with Cy5-labeled TNFα probe. RNA-EMSA was performed to examine whether the HuB-RRM3 domain influences the interaction between HuR and its target RNA.

**Fig. S5 The expression patterns of HuR and HuB in tumors**

**(A, B)** Expression of HuR and HuB in different tumors. According to the prediction results from an online database (www.aipufu.com), the expression levels of HuR (A) and HuB (B) in normal and tumor tissues were analyzed. BLCA, Bladder Urothelial Carcinoma. BRCA, Breast invasive carcinoma. CESC, Cervical squamous cell carcinoma and endocervical adenocarcinoma. CHOL, Cholangiocarcinoma. HNSC, Head and Neck squamous cell carcinoma. KICH, Kidney Chromophobe. LUAD, Lung adenocarcinoma. LUSC, Lung squamous cell carcinoma. PCPG, Pheochromocytoma and Paraganglioma. PRAD, Prostate adenocarcinoma. SARC, Sarcoma. SKCM, Skin Cutaneous Melanoma. STAD, Stomach adenocarcinoma.

**(C)** LPS stimulation promotes inflammation in mouse lung tissue and the cytoplasmic accumulation of HuR and HuB. After anesthetizing the mice, LPS was administered intranasally. Subsequently, lung tissue sections were prepared, and HE staining as well as immunohistochemical detection with HuR and HuB antibodies were performed to assess lung inflammation and the expression and localization of HuR/HuB. Scale bar, 50 μm.
